# Children's Embodiment of Non‐Human Virtual Hand Forms

**DOI:** 10.1111/desc.70131

**Published:** 2026-01-20

**Authors:** Hayley Dewe, Harry Brenton, Isabel Castelow, Emerald Grimshaw, Marco Gillies, Dorothy Cowie

**Affiliations:** ^1^ Department of Psychology Durham University Durham UK; ^2^ Bespoke VR Ltd. London UK; ^3^ Department of Computing Goldsmiths, University of London London UK

**Keywords:** development, embodiment, human forms, multisensory integration, training effects, virtual reality (VR)

## Abstract

Adults are known to identify their own body through a combination of multisensory cues and top‐down expectations regarding its form, while children may possess a more flexible body representation. Here we use virtual reality to test how children and adults use form cues to feel ownership over a virtual hand with novel, varying degrees of corporeality and how a sense of ownership of the hand and movement fluency with it may be trained. In Experiment 1, children (*N* = 40, 6–8 years) and adults (*N* = 45) experienced four virtual hand forms (Hand, hand with a missing Thumb, crab‐like Claw, Cross). Participants had to catch slowly moving virtual feathers while the virtual hand form moved in and out of synchrony with their own hand movements. In Experiment 2, we gave each child (*N* = 10, 6–9 years) and adult (*N* = 11) repeated experience with the Claw. Across studies we found that sensations of ownership over the virtual hand were facilitated by human‐like forms, movement synchrony, and short‐term training. For children only, we also found that human‐like forms maintained a strong facilitatory influence even when movement was asynchronous. Further, for children only, training improved movement fluency and increased the sense that the virtual form was a ‘tool' rather than a hand. We suggest that children's top‐down expectations regarding their body do not always interact with their multisensory inputs; their experiences are sharpened with training more than adults; and repeated short virtual experiences do not blur children's perceived distinction between the real and virtual self.

**Summary:**

- Both children and adults are sensitive to the corporeality of virtual hand forms, showing enhanced ownership for human‐like forms.
- While adults rely on concurrent movement synchrony and form, children treat them independently—maintaining some ownership for human‐like forms even when the movement was asynchronous.
- Short‐term training with a non‐human virtual form (crab‐like claw) increased ownership and improved movement fluency particularly for children.
- Compared to adults, children's embodiment of moving virtual hands reflects distinct processes—showing greater flexibility, independent cue use, and functional relevance.

## 1 Introduction

Embodiment encompasses sensations of ownership and agency over one's body, as well as feeling localised within it. These core features of human experience allow us to interact within the world and to feel distinct from other people and objects (Blanke and Metzinger 2009; Gallagher 2000). Such body representations arise through the integration of prior expectations about the body against incoming multisensory information, including visual, tactile, or proprioceptive cues.

In paradigms like the rubber‐hand illusion (RHI; Botvinick and Cohen 1998), one can manipulate multisensory signals to examine the underlying processes and constraints of own‐body representations and embodiment. In the RHI, individuals can embody a rubber hand that is in place of the individual's real (hidden) hand, provided that visuotactile signals (brush strokes) are delivered to the rubber and real hand and are temporally synchronous (Botvinick and Cohen 1998; Longo et al. 2008; Tsakiris and Haggard 2005). While this research has been largely dominated by adult work, in younger children (4–9 years) the RHI has revealed that while a sense of ownership is driven by synchronous visuotactile correlations (Cowie et al. 2013, 2016, 2018; Nava et al. 2017), simpler visual processes dominate for perceived hand location: viewing a rubber hand near the body prompts significant ‘proprioceptive drift’ (shift in perceived body location) toward the fake hand (Cowie et al. 2013, 2016; Filippetti and Crucianelli 2019).

While informative, these studies did not include the role of limb movement—since the illusion typically disappears as soon as the participant moves their own hidden hand. A sense of proprioception (movement and body position) plays a crucial part in understanding what drives embodiment (Corbetta and Snapp‐Childs 2009; Thelen and Smith 1996), and movement‐based exploratory processes have been observed in the first year of life (Delafield‐Butt et al. 2018; Rovee and Rovee 1969; Zoia et al. 2007), such as newborn reaches. In addition, infants show an ability to discriminate between discrepancies in visuomotor and visuotactile signals (Bahrick 2013; Bahrick and Watson 1985; Morgan and Rochat 1997; Rochat 1998); for example, 5‐month‐olds show preferential looking to a display of legs moving incongruently (asynchronous) to their own (Bahrick and Watson 1985). While the ability to detect multisensory discrepancies is present in newborns, little is known about how such processes contribute to children's own‐body representation and a sense of self (Bahrick 2013; Bremner and Cowie 2013).

Virtual reality (VR) offers an exciting opportunity to use virtually moving avatars or body parts to study embodiment and own‐body representation. Research has shown that visuomotor synchrony between a virtual body and an individual's own movements elicits stronger embodiment and is a key contributor to the effectiveness of VR experiences (Kilteni, Groten, et al. 2012; Sanchez‐Vives et al. 2010; Sanchez‐Vives and Slater 2005). Dewe et al. (2021) found increased embodiment (ownership, agency, location, and tool‐likeness ratings) for virtual hands in synchronous movement conditions in children aged 4–14 years, while Weijs et al. (2021) found movement synchrony influenced feelings of ownership but not agency in 8–12‐year‐olds.

Though the role of movement synchrony has been largely corroborated in these child samples, the effects of congruent visual signals, for example, the appearance of a virtual or fake body, are less clear. Understanding how incoming visual signals are interpreted against prior expectations or knowledge about the body is crucial for understanding the processes underlying own‐body representation. In RHI studies with adults, the strength of the illusion is reduced when the rubber hand is in an anatomically incongruent position or a non‐human texture/form (Gottwald et al. 2021; Tsakiris and Haggard 2005). Yet VR studies have revealed that adults can embody—to varying degrees—different virtual avatars, such as a body of a different gender (Petkova and Ehrsson 2008; Slater et al. 2010; Slater et al. 2019), age (Banakou et al. 2013), size (Piryankova et al. 2014) or skin colour (Maister et al. 2013, 2015; Peck et al. 2013). Further, adults have shown embodiment for modified, non‐corporeal human body parts/limbs, including an extended arm (Kilteni, Normand, et al. 2012) or a hand with six fingers (Hoyet et al. 2016), and remarkably, even for non‐human forms, including a body with a tail (Steptoe et al. 2013), animals (Ahn et al. 2016), or robot or zombie hands (Aymerich‐Franch et al. 2017; Lin and Jörg 2016).

These findings suggest there is a degree of malleability (or plasticity) of body representation among adults (Won et al. 2015), yet relatively little is known about the constraints or possibilities of body form in children's embodiment. Infants are sensitive to the configuration of their own legs (Morgan and Rochat 1997), and 6–7‐year‐olds do not embody a rubber hand placed in a different posture to their own hand (Gottwald et al. 2021). One study found that children aged 4–14 years will embody a non‐human virtual cross to some extent, but this was constrained by the tool‐like properties of the form and prior expectations of the body form (Dewe et al. 2021). Similarly, Weijs et al. (2021) found that while 8–12‐year‐olds felt less ownership for a virtual skeleton body than a human body, agency ratings were less affected by the likeness of human form. Together, these findings highlight discrepancies in our understanding of how children's embodiment is determined by body form and how this interacts with movement cues.

## 2 Experiment 1

The current study examined the effects and constraints of movement and human form on own‐body representation in children (age 6–8 years) and adults. At this age, children are known to be more visually captured in the RHI than adults. Coupled with the significant bodily growth at this age, we predicted that children might be more likely to embody non‐human hands than adults. To this end we created four virtual hand forms which systematically reduced in corporeality (a Hand, a hand missing a Thumb, a crab Claw, and a Cross). The task focused on the hands since research suggests a distinct developmental time course for hand embodiment (Cowie et al. 2013, 2016), and since they are a prominent visual feature, even from infancy (Smith et al. 2011). These forms were combined with both synchronous and asynchronous visuomotor signals, which we predicted would be important at both ages.

Wearing an *Oculus Rift* headset and holding an *Oculus Touch* controller, participants were asked to ‘catch’ virtual falling feathers under different movement conditions and using different virtual hand forms. It was situated in a vibrant funfair environment as per Dewe et al. (2021) and designed to be an engaging task for young children. We wanted to encourage controlled and slow movements which reinforced focus on the virtual hands and therefore used small feathers as targets since these fell slowly and could be manipulated (i.e., flicked, spun, or pushed) by the participant after being caught. The brightly coloured feathers dropped one at a time before disappearing, providing an exciting and easy cue to the next movement. Lastly, the expectation of lightness regarding the feathers made the lack of haptic feedback feel acceptable to users.

### 2.1 Method

#### 2.1.1 Participants and Design

We recruited 45 adults aged 18–39 years (*M* = 21 years, SD = 4.73, 34 females, 38 right‐handed) who were undergraduate students from Durham University and 45 children aged 6–8 years (*M* = 7 years, SD = 0.83, 25 females, 39 right‐handed) from the urban area of Durham, United Kingdom. In this area approximately 96.8% of this population identify as White (including White British, White Irish, and other White backgrounds; Office for National Statistics, 2021). All participants had normal or corrected‐to‐normal vision and no known neurodivergence, or sensory or motor conditions. The sample was slightly larger than recommended by a priori power calculations in G*Power 3.1. Based on a previous similar VR task (Dewe et al. 2021) and comparable work exploring the effects of movement in children (Cowie et al. 2013) and movement and virtual forms in children and adults (Weijs et al. 2021), a medium‐to‐large estimated effect size ($n_{p}^{2}$’s < 0.115) was chosen for the effects of movement with *α* error probability = 0.05 and power = 0.8, yielding a sample size between 18 and 38 participants, and a small‐to‐medium estimated effect size ($n_{p}^{2}$’s < 0.06) with *α* error probability = 0.05 and power = 0.8 for the effects of form, and interaction effects between movement, form and age yielded a sample size between 24 and 46 participants. Our final sample sizes (*n* = 45 for adults, *n* = 40 for children), explaining drop‐out rates, are shown in Table S1. This study was approved by the research ethics committee at Durham University.

#### 2.1.2 Catching Feathers Task

**FIGURE 1:**
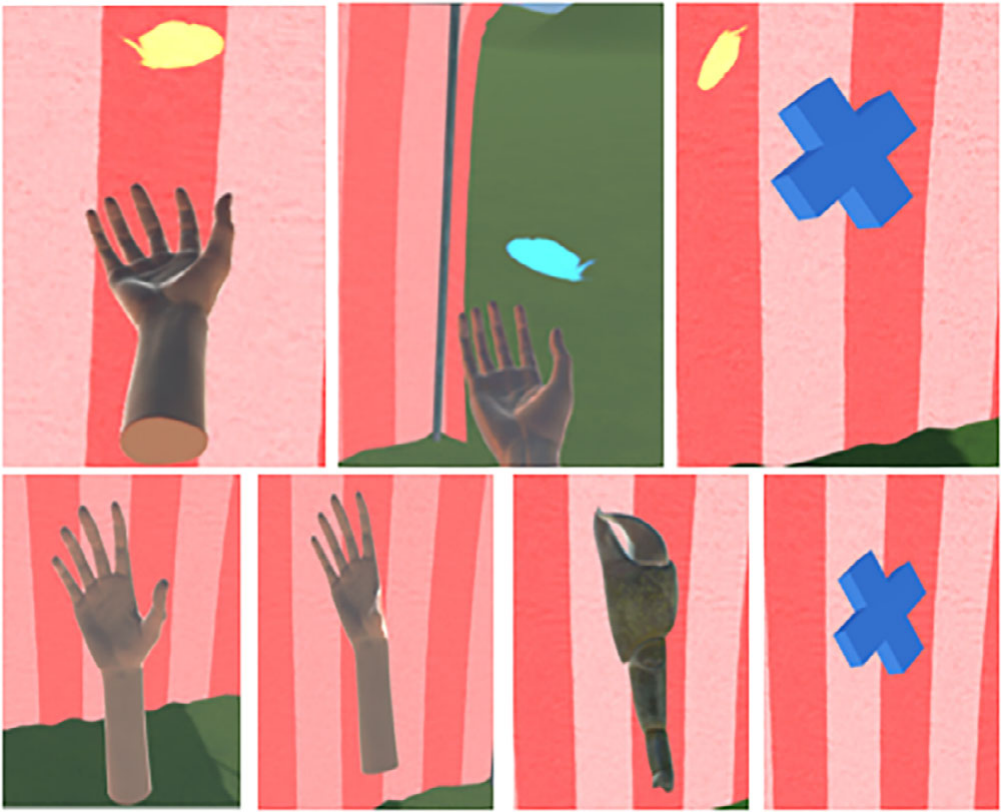
Images of the Catching Feathers VR task. Top row: examples of two forms (the Hand and Cross) catching falling feathers. Bottom row: the four virtual forms (set here to size 15 cm), from left to right: a human Hand (adult size), the Thumb (hand with missing thumb), the Claw and the Cross. All virtual forms are in the position with the participants palm facing upwards.

The Catching Feathers task was created as a custom‐built virtual funfair environment, designed in Unity (Unity Technologies, San Francisco, CA, USA) through an *Oculus Rift* head‐mounted display (HMD; Oculus, Menlo Park, CA, USA). To track movement, participants held an *Oculus Touch* controller in their right hand while standing in front of a virtual circus tent and were asked to ‘*catch’* the brightly coloured feathers as they fell from the top of the tent down in front of them (Figure 1). The feathers dropped every 3 seconds at random from 1 of 4 locations on a straight line above the participant's head. Only one feather was visible at a time, and each took 4 seconds to fall before disappearing (the feathers disappeared whether they were caught or missed with the virtual hand).

During the task, all participants used each of the four virtual hand forms that varied in corporeality (Figure 1): a human hand (Hand) that was either a child or adult hand size, a hand with a missing thumb (Thumb), a claw (Claw) and a non‐human 3D cross (Cross). The forms were designed to represent a gradient of human hand corporeality, ranging from human‐like hands to progressively altered structures (from human‐like to the crab‐like claw to the abstract cross). All forms were taken from the VR hands and FP Arms Pack by NatureManufacture^1^. The human forms were realistic representations of a human hand with simplified light skin colour/tone, corresponding to much of the current sample, while the Claw was dark grey in colour and positioned in a pincer grip, and the Cross was a vibrant blue colour. The forms were scaled to each participant's hand size, which was the distance (cm) between the tip of the middle finger and the crease of the wrist of the right hand. They were disconnected at the wrist. Instructions were: ‘*You will be placed in a fairground scene and there will be a red and white stripy tent in front of you where the multicoloured feathers will fall. You are required to move the virtual hand beneath the feathers, allowing the feathers to land on the virtual hand. Your task is to catch as many feathers as possible before they disappear by balancing them at least once on the virtual hand*’.

There were two movement trials during the task, where the virtual hand moved in temporal synchrony in real time with the participant's own hand movements or asynchronously, where participants had no control over the hand's movements. In synchronous movement conditions, the participant's hands were tracked by position and rotation (six degrees of freedom) directly onto the virtual forms (Hand, Thumb, and Claw) while the Cross was rotated by 45°. There was no other form of hand tracking (the fingers did not move). Participants held the controller with their palm facing upwards and fingers outstretched so the virtual and real fingers (of the human hands) approximately matched in posture, which also simulated how one would typically catch an object in real life. For asynchronous movement trials, prior recordings of the participants’ hand movements were taken during a ‘practice’ trial of feather catching and played back in place of the live hand tracking data. This meant that during asynchronous trials, the virtual hand moved as per the movements of the prerecording and did not respond to the participant's movements in real time, and was spatially and temporally incongruent from their own hand.

#### 2.1.3 Embodiment Ratings

After each trial of feather catching, participants responded to five embodiment‐related questions and a control question for each virtual form (Table 1). These questions pertained to Ownership, Agency, Appearance^2^, Task‐Effectiveness (i.e., whether the form felt specifically like their own hand for completing the task effectively), and Tool‐Likeness (i.e., whether the form felt specifically more like a tool or object that facilitated the feather‐catching task). Questionnaire measures were developed and based on a previous comparable VR task with children (Dewe et al. 2021) and previous measures of embodiment with children (Cowie et al. 2013, Cowie et al. 2018, 2016; Gottwald et al. 2021; Keenaghan et al. 2020; Keenaghan et al. 2022; Weijs et al. 2021) and adults (Braithwaite et al. 2017; Gonzalez‐Franco and Peck 2018; Longo et al. 2008; Peck and Gonzalez‐Franco 2021). There were subtle but intentional differences between (Q1) Ownership and (Q4) Task Effectiveness: to compare an established measure of direct bodily ownership with a question specifically targeting ownership during the action‐based task (catching feathers).

**TABLE 1:** Embodiment questions used in Experiments 1 and 2.

	QUESTION	COMPONENT
	Sometimes during the game, it felt as if:	
Q1	the virtual hand/claw/cross was my hand	Ownership
Q2	I could control the virtual hand/claw/cross as if it was my own hand	Agency
Q3A	my real hand had a shape or texture like the virtual hand/claw/cross that I saw	Appearance
Q3B	the virtual hand/claw/cross looked like my hand in terms of shape, size or texture	
Q4	the virtual hand/claw/cross was my own hand, catching the feathers	Task‐Effectiveness
Q5	the virtual hand/claw/cross was just a tool I used to catch the feathers	Tool‐Likeness
Q6	the virtual hand/claw/cross changed shape during the game	Control

*Note*: For analysis, Q3A and Q3B were averaged to formed ‘Q3’ data, which is presented in all figures and analysed along with the other questions.

Like previous studies (Dewe et al. 2021; Keenaghan et al. 2020), we included a Control question to ensure that participants were answering appropriately; here this question asked whether the virtual form changed in shape during the task (it did not). Participants answered all questions in VR using the controller, which emitted a virtual laser beam to indicate their response (a circle) along a 7‐point Likert scale ranging from 0 (‘*No, not at all*’) to 6 (‘*Yes, lots and lots*’). The experimenter simultaneously read the questions aloud twice to ensure understanding and reminded participants they could answer anywhere along the scale (Dewe et al. 2021; Cowie et al. 2013).

#### 2.1.4 Procedure

Participants entered the virtual environment wearing the *Oculus Rift* HMD and holding the controller in their right hand. First, they completed a brief training session on the questionnaire response scale and used the virtual slider to rate foods and homework. Next, they completed one block of nine trials, each trial lasting 60 seconds. This included one practice trial of feather catching (which was screen‐recorded and used for asynchronous movement trials), four synchronous movement trials with each of the four virtual forms (Hand, Thumb, Claw and Cross) and four asynchronous movement trials with each of the four forms. All movement trials were randomised, and after each movement trial, participants answered the embodiment questions in VR (Table 1) for the trial they had just experienced. The questionnaire was read aloud by the experimenter, and participants answered on the virtual scale placed in front of them. The experiment took approximately 20 minutes.

### 2.2 Results

The ordinal data were first transformed via an Aligned Rank Transformation (ART; Wobbrock et al. 2011) as per previous comparable studies (Cowie et al. 2018; Dewe et al. 2021; Gottwald et al. 2021). This procedure aligns and ranks the raw data before they are subjected to analysis, allowing examination of main effects and interactions of ordinal data. For each question (see Table 1), we analysed the transformed data using three‐way, 2 (movement: synchronous, asynchronous) × 4 (Form: Hand, Thumb, Claw and Cross) × 2 (Group: Children, Adults) ANOVAs. Since we predicted a priori that the hand would be the most embodied, followed by Thumb, Claw and then Cross, we report linear contrasts for form. All figures (additionally for Experiment 2) present medians and IQRs, overlaid with individual data points, and were created in R using ggplot2 package (R Core Team 2024; Wickham 2009).

**FIGURE 2:**
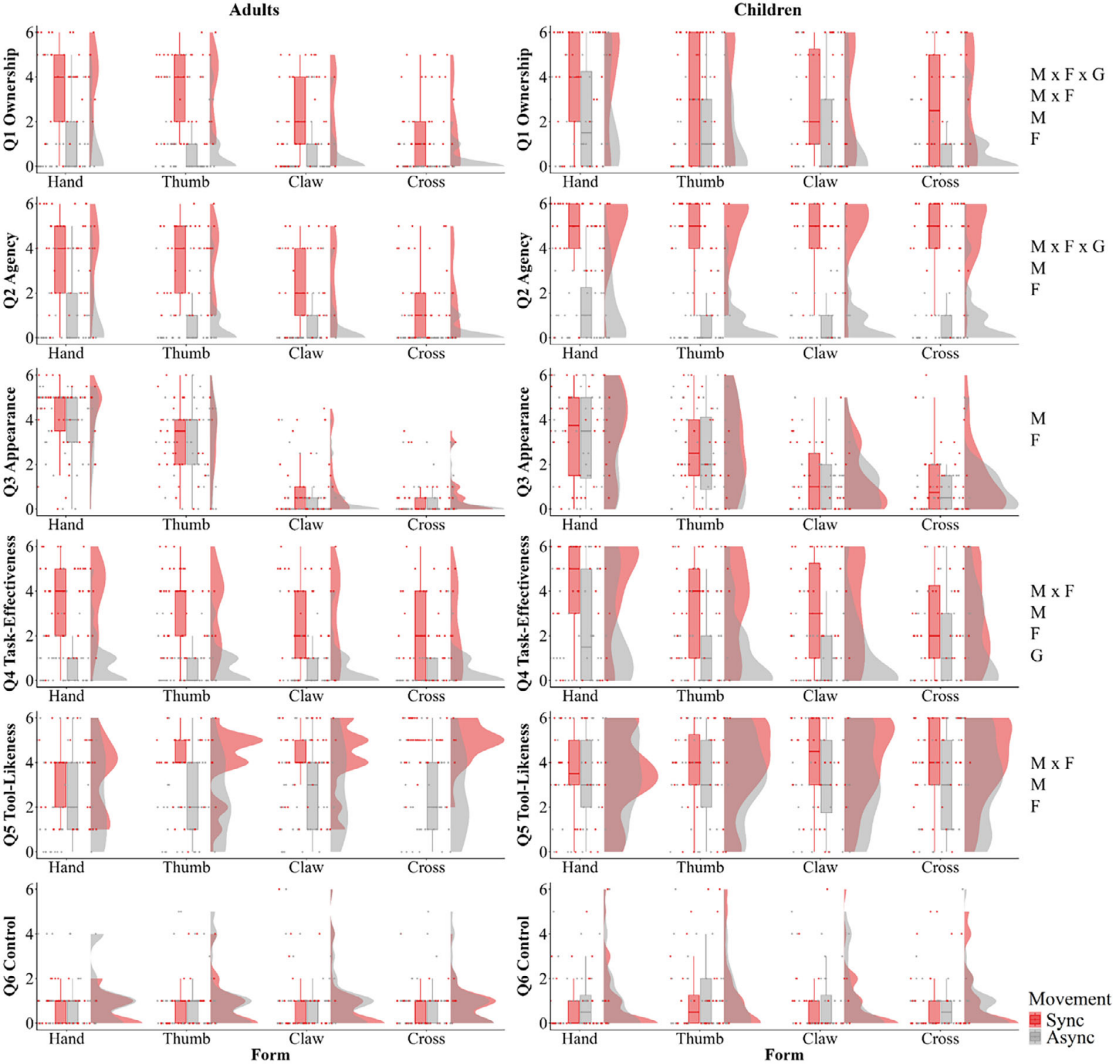
Questionnaire results of Experiment 1 for both adults and children for each question rating. From top to bottom row: ratings of Ownership (Q1), Agency (Q2), Appearance (Q3), Task‐Effectiveness (Q4), Tool‐Likeness (Q5), and Control (Q6, Table 1) for adults (left column) and children (right column). The text on the right side of the figure indicates significant effects for each question: M = Movement, F = Form, G = Group.

First, we analysed ratings from the Control question (Table 1, Q6; Figure 2) to ensure both children and adults were answering appropriately, and similarly. Responses to this question were low, and ratings were similar between adults and children. Mann–Whitney U tests (Bonferroni corrected for multiple comparisons) revealed no significant differences between groups for any of the form/movement combinations (all *p*’s > 0.375, all *U*’s > 805.50).

#### 2.2.1 Ownership Ratings

For Ownership (Table 1, Q1; Figure 2), there was a three‐way interaction between movement, form and group, *F*(1, 83) = 4.483, *p* = 0.037, $\eta_{p}^{2}$ = 0.05. There was a two‐way interaction between form and movement, *F*(1, 83) = 15.957, *p* < 0.001, $\eta_{p}^{2}$ = 0.16, but no other two‐way interactions (*p*’s > 0.05). There was a main effect of movement, *F*(1, 83) = 146.884, *p* < 0.001, $\eta_{p}^{2}$ = 0.64, and form, *F*(1, 83) = 88.801, *p* < 0.001, $\eta_{p}^{2}$ = 0.52, but not group (*p* > 0.05). To break down the three‐way interaction, we looked at each age group separately at the interaction between form and synchrony. We found no significant interaction for children (*p* = 0.834), but a significant interaction for adults, *F*(1, 44) = 42.422, *p* < 0.001, $\eta_{p}^{2}$ = 0.49.

Breaking down this interaction in adults, there were effects of form for both synchronous, *F*(1, 44) = 97.823, *p* < 0.001, $\eta_{p}^{2}$ = 0.69, and asynchronous, *F*(1, 44) = 15.778, *p* < 0.001, $\eta_{p}^{2}$ = 0.26, conditions. We took a best‐fit slope across the four forms for the synchronous and asynchronous movement conditions, where 1 reflected the most human‐resemblance (Hand) and 4 reflected the least similar (Cross). On average, slopes were significantly steeper for synchronous (−0.75) than for asynchronous (−0.31) movement (*t*(44) = 4.48, *p* < 0.001, *d* = −0.67). This suggests that adults' sense of ownership over a virtual hand is more strongly influenced by the interaction between form and movement synchrony, with human‐like forms leading to greater ownership, particularly in synchronous movement conditions. In contrast, children's sense of ownership was not affected by the interaction between form and synchrony, suggesting that their sense of ownership may be less dependent on these factors.

#### 2.2.2 Agency

For Agency ratings (Table 1, Q2; Figure 2), there was a three‐way interaction between movement, form and group, *F*(1, 83) = 4.895, *p* = 0.030, $\eta_{p}^{2}$ = 0.06. There were no two‐way interactions (all *p*’s > 0.05). There was a main effect of movement, *F*(1, 83) = 523.181, *p* < 0.001, $\eta_{p}^{2}$ = 0.86, and form, *F*(1, 83) = 7.20, *p* = 0.009, $\eta_{p}^{2}$ = 0.08, but not group (*p* > 0.05). To break down the three‐way interaction, we looked at each age group separately at the interaction between form and synchrony. We found no significant interaction for children (*p* = 0.604), but a significant interaction for adults, *F*(1, 44) = 8.85, *p* = 0.005, $\eta_{p}^{2}$ = 0.17. There was an effect of form for the synchronous, *F*(1, 44) = 4.16, *p* = 0.047, $\eta_{p}^{2}$ = 0.09, but not asynchronous (*p* > 0.05) conditions. It suggests that, like ownership, adults’ sense of agency is influenced by the interaction between form and movement synchrony, with human‐like forms and synchronous movement leading to higher agency. In contrast, children's sense of agency appears less dependent on these factors. To better characterise these effects, for the adults, as for Q1 Ownership, we compared the best‐fit slope across the four forms for the synchronous and asynchronous movement conditions (from 1 = Hand to 4 = Cross). A Wilcoxon signed‐rank test indicated that the slopes differed significantly between the synchronous (*M* = −0.13) and asynchronous (*M* = 0.01) conditions (*Z* = −2.26, *p* < 0.020, *r_rb_* = −0.40, reflecting a moderate effect). This suggests that adults showed some downward trend from the most human‐like hand to the least under the synchronous movement condition only, while the slope in the asynchronous movement condition was extremely close to zero.

#### 2.2.3 Appearance

For Appearance ratings, the score was derived from averaging the two appearance questions (Table 1, Q3A and Q3B; Figure 2), as they both measured the same concept but were phrased slightly differently to ensure participant understanding^3^. There were no significant interactions (all *p*’s > 0.05). There was a main effect of movement, *F*(1, 83) = 5.75, *p* = 0.019, $\eta_{p}^{2}$ = 0.06, and a main effect of form, *F*(1, 83) = 316.06, *p* < 0.001, $\eta_{p}^{2}$ = 0.79, but not group (*p* > 0.05). This suggests that Appearance ratings for both adults and children were influenced by form and movement, with human‐like forms and synchronous movement conditions receiving the highest ratings. There were no differences in Appearance ratings between adults and children.

#### 2.2.4 Task‐Effectiveness

For Task‐Effectiveness (Table 1, Q4; Figure 2), there was a two‐way interaction between movement and form, *F*(1, 83) = 19.00, *p* < 0.001, $\eta_{p}^{2}$ = 0.19. All other interactions were non‐significant (all *p's* > 0.05). There were main effects of movement, *F*(1, 83) = 134.07, *p* < 0.001, $\eta_{p}^{2}$ = 0.62; form *F*(1, 83) = 42.01, *p* < 0.001, $\eta_{p}^{2}$ = 0.34; and group, *F*(1, 83) = 7.82, *p* = 0.006, $\eta_{p}^{2}$ = 0.09. This suggests that ratings were influenced by both form and movement, with human‐like forms under synchronous movement leading to higher ratings. There was no interaction with group; however, the main effect of group indicates children rated the forms as more effective than adults in catching the feathers overall.

#### 2.2.5 Tool‐Likeness

For Tool‐Likeness (Table 1, Q5; Figure 2), there was a two‐way interaction between movement and form, *F*(1, 83) = 6.97, *p* = 0.010, $\eta_{p}^{2}$ = 0.08. All other interactions were non‐significant (all *p's* > 0.05, though the interaction between form × group was borderline, *p* = 0.052, with a small, approaching medium effect size $\eta_{p}^{2}$ = 0.05). There were main effects of movement, *F*(1, 83) = 30.28, *p* < 0.001, $\eta_{p}^{2}$ = 0.27, and form, *F*(1, 83) = 9.28, *p* = 0.003, $\eta_{p}^{2}$ = 0.10, but not group (*p* > 0.05). Ratings were influenced by both form and movement, with human‐like forms under synchronous movement leading to higher ratings. The borderline interaction between form and group suggests there may be subtle differences in how adults and children rated the Tool‐Likeness of the form (used as an instrument).

## 3 Experiment 2

While Experiment 1 examined the embodiment of non‐human virtual hand forms during one short session, in Experiment 2 we examine how this embodiment may grow or change with training. Previous work has shown that motor performance improved after short‐term training with a virtual hand form (Argelaguet et al. 2016). Similarly, after five daily sessions using a robotic third thumb, feelings of ownership as well as motor responses were shown to improve (Kieliba et al. 2021). In the present study we specifically focus on how training over several days might impact upon the embodiment of a non‐human virtual hand form, namely, the Claw used in Experiment 1. The non‐human texture and shape of the Claw contrast with its familiar human‐like pincer grip; levels of Ownership in Experiment 1 were intermediate. We therefore chose this form as one that might benefit from short‐term training, which was spread across three sessions over a 10‐day period.

As in Experiment 1, embodiment was assessed by questionnaire. We also introduced additional tests of reaction to a virtual threat and movement fluency. These were measured during a new section of experience in which users moved the Claw systematically back and forth over a mildly threatening virtual object (a cactus). We hypothesised that with training, participants would exhibit greater vertical clearance (i.e., a larger margin of error) when moving the virtual hand over the threatening virtual cactus, a signature of the defensive or protective reactions argued to be a hallmark of body ownership (de Vignemont 2023a). Indeed, participants have been shown to give heightened skin conductance responses to a threatened virtual hand (Tieri et al. 2015) and to move their virtual hands with extreme care over a virtual spinning saw (Argelaguet et al. 2016). As in previous studies, we also hypothesised that fluency of control would improve with training (Argelaguet et al. 2016; Kieliba et al. 2021). This may be at least a ‘weak’ signature of motor embodiment in the sense that the properties of the virtual form are processed in the same way as the properties of the user's own body (de Vignemont 2018, 2023b). In this study we assessed movement fluency as the movement speed of the hand across the virtual objects.

In addition to considering these measures of embodiment, we tested whether training on this form might generalise to other forms (the Hand and Cross forms from Experiment 1) and other movement types (the asynchronously moving Claw). The hypothesis that training one form might generalise to another arises from a desktop rubber hand illusion study which found that training with one form (a fake hand) allowed embodiment of another (a box), as the user's conception of their body gradually shifted to enable embodiment of non‐corporeal objects (Hohwy and Paton 2010), and is in line with a predictive coding framework where priors can be updated (Apps and Tsakiris 2014). We predicted more ready generalisation towards new forms than towards asynchronous movement, which strongly blocked embodiment in Experiment 1.

In common with Experiment 1, based on previous work on desktop bodily illusions (Cowie et al. 2013; Filippetti and Crucianelli 2019), we predict a higher degree of plasticity in body representation for children than for adults. This would manifest as the above measures changing with greater magnitude for children than for adults. Given the lack of previous comparable data, it is difficult to specify whether these changes might happen over the same time period in both groups or over a longer period for children.

In sum, we hypothesised that as training progressed, participants would show increased subjective embodiment of the virtual Claw, more prominent responses to the virtual threat, and higher movement fluency. Further, we hypothesised that embodiment might generalise from the Claw to the other forms presented and that children might show greater training effects than adults.

### 3.1 Method

#### 3.1.1 Participants and Design

**FIGURE 3:**
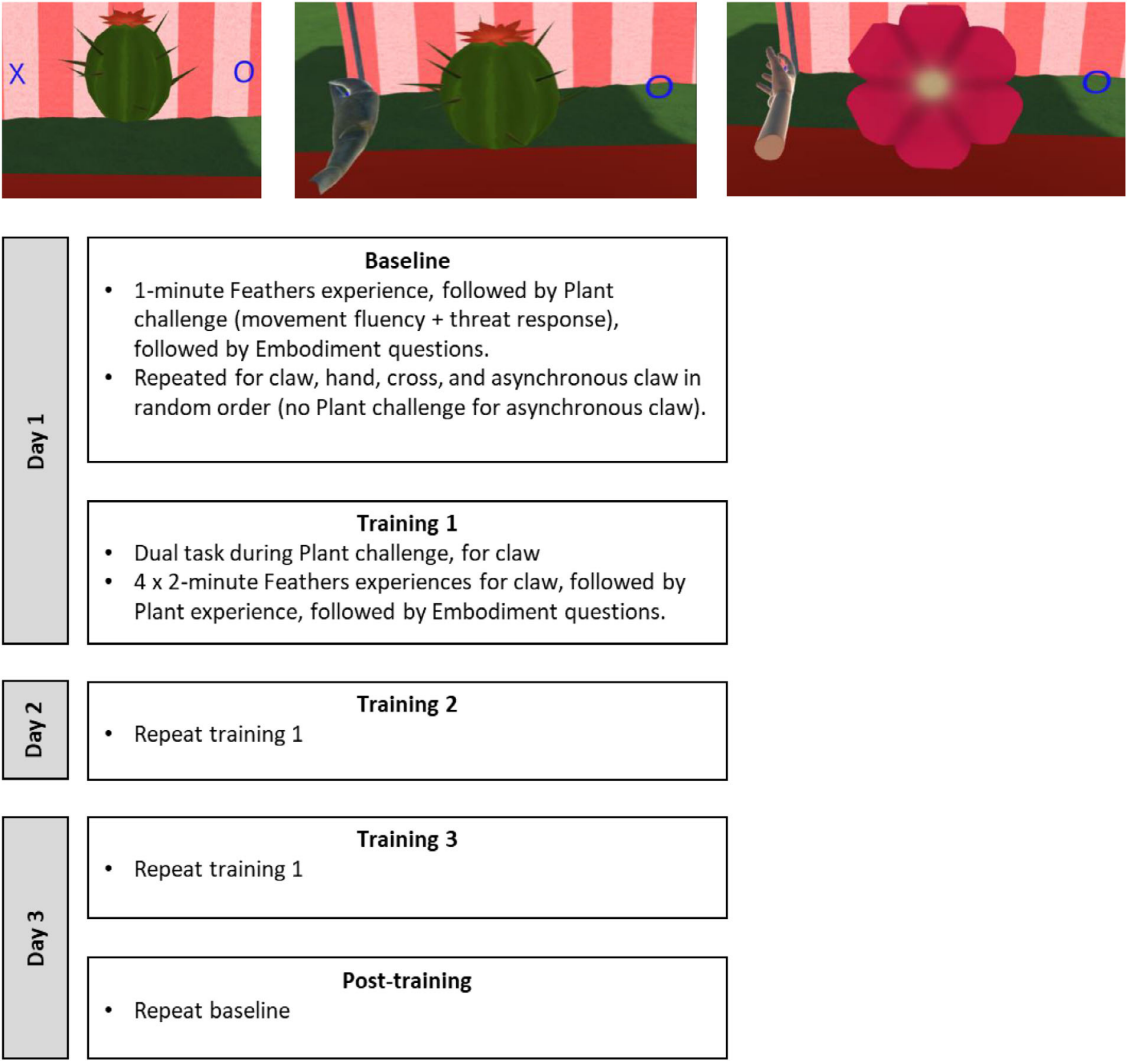
Images of the Plant Challenge and a structure of the training sessions. Top from left to right: Participants moved their hand from X‐O‐X over a cactus (threat condition) or flower (non‐threat condition). Bottom: structure of the 3‐day VR Claw training sessions.

We recruited 12 adults aged 18–21 years (*M* = 20 years, SD = 0.90, 11 females, 11 right‐handed) who were undergraduate students from Durham University and 11 children aged 6–9 years (*M* = 8 years, SD = 0.90, 5 females, 10 right‐handed) recruited from the same area as described in Experiment 1. Due to the intensive multi‐day repeated‐measures protocol with children, we used a smaller sample to ensure feasibility and retention. Our design was informed by previous body ownership studies with children (∼15 per group; Cowie et al. 2016; Filippetti and Crucianelli 2019). Although our sample was slightly smaller, the increased number of repeated measures (five per participant) provided sufficient power to detect changes in ownership. Our final sample sizes, which varied slightly across measures, are explained in Table S1. All participants had normal or corrected‐to‐normal vision and no known neurodivergence or sensory or motor conditions. Each participant completed 3 testing days within a 10‐day period (Figure 3). This within‐subjects design tested the impact of training on performance. Ethical approval was gained from Durham University ethics committee.

#### 3.1.2 Catching Feathers Task and Plant Challenge

The Catching Feathers task was as described in Experiment 1, with the following few exceptions: (i) Participants used only the virtual Hand, Claw, and Cross forms, the Thumb form was removed. (ii) We removed questions on Appearance and Task‐Effectiveness (Table 1, Q3A, Q3B and Q4). Additionally, we introduced the ‘Plant challenge’ to measure threat response and movement fluency (Figure 3). Movement over a spiky virtual cactus was compared with movement across a similarly proportioned, but non‐threatening, virtual flower. During this challenge, the controller's position and movement were recorded by Unity. The movement data were analysed in MATLAB, which calculated (i) threat response, operationalised as the vertical clearance of the hand above the centre of the plant object, and (ii) movement fluency, operationalised as total movement duration for the X‐O‐X sequence. Instructions were: *‘A plant will appear in front of you, and you will see an X and an O at either side. Place your hand on the X; move it over the top of the plant to the O; and then back to the X, again over the top of the plant*’.

Each time participants took part in the Plant Challenge they completed 4 conditions: plant single‐task, plant dual‐task, cactus single‐task, and cactus dual‐task. Single tasks were as described above. Dual‐task conditions required participants to perform a short numerical task during the movement: slower movement here could indicate some lack of automaticity (Witteveen et al. 2012; Kieliba et al. 2020). Participants were read a set of 8 numbers as they moved from X to O, which they repeated while moving back from O to X. However, experimental error meant the total movement time included a period where the participant could not move at their own pace, and so dual‐task performance was not analysed further.

#### 3.1.3 Procedure

Participants first took part in some short tests of vision and balance, not analysed here. Across 3 days, participants then took part in the Catching Feathers task and the Plant Challenge as described in Figure 3. This design assessed embodiment of the Hand, Cross, and Claw at Baseline and Post‐test (post‐training), each time using three measures (questionnaire, threat response, movement fluency). For training, we assessed embodiment across three sessions where participants received movement experience with only the virtual Claw form. Note: there was no plant challenge for the asynchronous Claw condition, as participants could not control it; they instead answered the questionnaire directly after the Catching Feathers task. The experiment took approximately 20 minutes each day over 3 days.

### 3.2 Results

#### 3.2.1 Questionnaire

**FIGURE 4:**
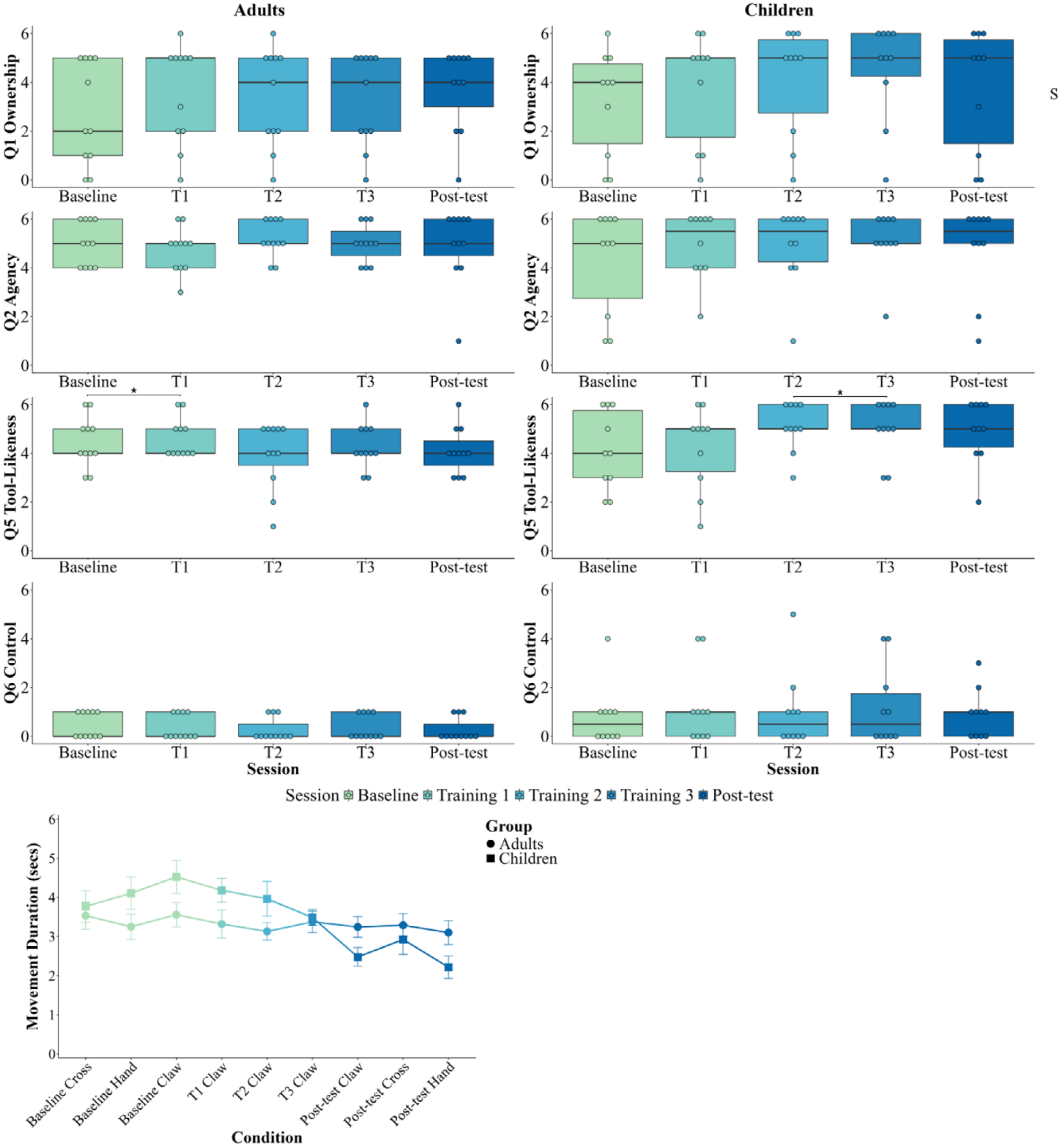
Questionnaire results (top) and average movement duration (bottom) for both adults and children across each training session in Experiment 2. Top figure: questionnaire ratings of Ownership (Q1), Agency (Q2), Tool‐Likeness (Q5), and Control (Q6, Table 1) for adults (left column) and children (right column). The text on the right side of the figure indicates significant effects across groups for each question: S = Session, and * indicates *p* < 0.05. Bottom figure: average movement duration (seconds) for adults and children for the Baseline and Post‐test sessions with the three forms (Cross, Hand, asynchronous Claw), and the three training sessions (T1–T3, respectively) with the synchronous Claw.

Figure 4 presents medians and IQRs for Ownership, Agency, and Tool‐Likeness and Control ratings (Table 1, Q1, Q2, Q5, Q6) during each training session for adults and children. Since the Control ratings showed no significant effects of age or session, we have no reason to believe that response bias significantly affected the responses given to the other questions. We therefore examined embodiment of the Claw using ART‐corrected 2 (group: children, adults) × 5 (training session: Baseline, Training 1, Training 2, Training 3, Post‐test) ANOVAs (see Experiment 1).

For Ownership, there was no significant effect of group or interaction with session (*F*’s < 1.285, *p*’s > 0.283, $\eta_{p}^{2}$’s < 0.06), but a main effect of session (*F*(2.3,43.6) = 3.06, *p* = 0.050, $\eta_{p}^{2}$ = 0.14, corrected for Sphericity violation). To understand the pattern of learning across sessions, we used Bayesian Wilcoxon Signed Rank *t*‐tests to supplement a difference contrast from each training session to the next. Overall, there was a slight gradual increase (trend) across sessions and a significant increase from Baseline to Training 1 only (*p* = 0.011, BF^10^ = 9.56, moderate evidence for the alternative hypothesis). There were no further significant increases between the remaining training sessions (*p*’s > 0.174, BF^10^’s = 0.27–1.02, indicating anecdotal evidence).

Agency remained high throughout, with no significant effects (*F*’s < 0.727, *p*’s > 0.529, $\eta_{p}^{2}$’s < 0.04). The Tool‐Likeness ratings showed no main effects (*F*’s < 1.063, *p*’s > 0.315, $\eta_{p}^{2}$’s < 0.05), but an interaction (*F*(4, 76) = 5.209, *p* < 0.001, $\eta_{p}^{2}$ = 0.22) where follow‐up Friedman tests revealed that children (*Χ^2^* (4) = 10.09, *p* = 0.039) but not adults (*Χ^2^* (4) = 7.56, *p* = 0.109) gave higher ratings of Tool‐Likeness as sessions progressed. We used Bayesian Wilcoxon Signed Rank *t*‐tests to supplement a difference contrast from each training session to the next for children and adults. Children showed a significant increase from Training 2 to Training 3 only (*p* = 0.028, BF^10^ = 4.90, moderate evidence for the alternative hypothesis). Adults showed a significant increase from Baseline to Training 1 only (*p* = 0.018, BF^10^ = 10.97, moderate‐strong evidence for the alternative hypothesis). There were no further significant increases between the remaining training sessions for either group (*p*’s > 0.097, BF^10^’s = 0.39–0.89, indicating anecdotal evidence).

To further characterise the effect of training and age, we conducted at each age a best‐fit slope analysis which examined changes in Tool‐Likeness ratings across the five sessions. A Mann–Whitney *U* test revealed that these slopes differed significantly between adults (*M* = −0.11) and children (M = 0.24), *U* = 89.00, *p* < 0.017, *r_rb_* = 0.62, indicating a moderate‐to‐large effect. This suggests that children's Tool‐Likeness ratings increased as their training progressed, whereas adults showed little to no change across training sessions.

To assess generalisation to other forms (Hand, Cross and asynchronous Claw), we examined Baseline versus Post‐test ratings using ANOVA with factors form and group. Feelings of Ownership, Agency and Tool‐Likeness over these forms did not change following training with the Claw, nor were they affected by age (Table S2).

#### 3.2.2 Threat Response

We examined avoidance behaviour as the virtual hand form moved over the cactus and the flower obstacles. At Baseline, adults (Table S3) raised the Claw an average of 3.23 cm higher above the cactus than the flower (*t*(11) = 2.97, *p* = 0.013, *d* = 3.88, 95% CI [0.18, 1.51]). This increased caution was not seen in children, where responses were similar for the two plant types (*p* = 0.362). For adults, therefore, the difference in hand height over the cactus and flower was an appropriate index of perceived threat, while for children it was not. For adults, this difference score did not change with training session (*p* > 0.200), indicating that the perceived threat did not diminish with practice. Likewise, it did not change from Baseline to Post‐test for the Hand or Cross forms (*p*’s > 0.700). As noted, children took care to reach over both plant types. The peak height reached by their virtual hand relative to the cactus was unaffected by session (*p* > 0.60) or from Baseline to Post‐test (*p*’s > 0.15, Table S4). Overall, therefore, we observed no experimental effects on participants’ consistent patterns of caution as they crossed the virtual obstacles.

#### 3.2.3 Movement Fluency

We examined control of the Claw by testing how movement duration changed across sessions (Figure 4). A linear mixed‐effects model (LMER, R Core Team 2024) was used, with fixed effects for session and group and random effects for participants to account for repeated measures. With one missing value in each group due to failed recording, the analysis revealed a group‐by‐session interaction (*t*(19) = −3.94, *p* < 0.001). While adults showed no significant effect of session (*p* = 0.274), children did (*t*(8) = −4.60, *p* = 0.002), with session 3 (*p* = 0.012) and post‐test (*p* < 0.001) being of significantly lower duration than Baseline. This improved control (with the Claw) generalised to the Hand and Cross for children: both were faster than at Baseline (Hand, *t*(7) = 3.96, *p* = 0.005, *d* = 1.40, 95% CI [0.38, 2.38]; Cross, *t*(8) = 2.97, *p* = 0.018, *d* = 0.99, 95% CI [0.16, 1.78]). For adults, in contrast, movement duration for these forms did not change from Baseline to Post‐test (*p*’s > 0.356).

## 4 General Discussion

We compared children's and adults’ embodiment of non‐human forms through an initial experiment with four forms, as well as a second experiment to examine the impact of training with one form. The results contribute to our knowledge of the sensory and cognitive factors underpinning children's embodiment. Further, although many VR platforms such as Meta recommend that their devices should not be used by children under 13 years, there is considerable evidence that children are using VR regularly. For example, a 2024 survey (Piper Sandler 2024) found that 33% of US teens own a VR HMD and that a large proportion of users of social VR platforms are children (Maloney et al. 2020 March). Despite this prevalence of children among VR users, there is still a lack of a strong evidence base on how VR impacts children differently from adults (Bexson et al. 2024), including on the impacts of multisensory cues and embodiment.

A sizeable literature shows that adults can embody non‐human virtual hands to some degree (Mottelson et al. 2023). However, it is also clear that constraints apply; there is an uncanny valley for mildly unrealistic hands, and larger distortions like a robot or wooden hand are harder to embody (Lin and Jörg 2016). Our first experiment replicated this pattern, showing that adults can accept distortions such as a missing thumb with relative ease, while the more extreme blue cross was not well tolerated. The fact that structural distortions like the Thumb were easier to accept than featural distortions of texture (Claw) or colour (Cross) adds to a need for systematic investigation of which aspects of corporeality are most key for adult embodiment.

Static desktop rubber hand illusion studies indicate that children may be subject to a different pattern of sensory weightings or top‐down constraints than adults. For example, children have been shown as more likely than adults to embody a viewed static rubber hand (Cowie et al. 2013; Filippetti and Crucianelli 2019) and to accept distortions of its form (Preston et al. 2015) or size (Cowie et al. 2022; Keenaghan et al. 2022). However, there has been little work on how this implied plasticity in body representation plays out in the context of a moving hand. To examine this, we employed VR to create a multisensory, dynamic context where participants control a virtual hand differing in form from one's own. Further, visuomotor synchrony was varied so that the virtual hand's movement was synchronous or asynchronous with the participant's own. One previous study with children showed that this synchrony cue is important and that ownership was low for a very non‐human cross form (Dewe et al. 2021). However, there was no direct comparison to an adult sample and, crucially, no examination of any more nuanced effects of form. This is important both for a full understanding of the cues governing embodiment and because in virtual experiences children may increasingly be exposed to an array of non‐human bodies (Bailey and Bailenson 2017).

The present results address this gap in the literature, showing a graded drop‐off in ownership with decreasing human‐likeness of virtual hand forms, not only for adults but also for children. Like adults, children are sensitive to subtle changes in hand form, suggesting that form operates as a nuanced cue to embodiment. In applied terms, children in embodied VR experiences can be expected to experience a range of forms as their own, within limits of corporeality. The forms we used had a varied combination of material (textural, colour) changes as well as structural (anatomical) differences. We specifically find that minor structural changes (missing Thumb) have less effect than major structural changes (Claw, Cross): when an almost‐anatomical configuration is preserved, embodiment approaches that of a human hand (see also Weijs et al. 2021). However, the Claw form and blue Cross also had changes to texture and colour. We suggest, therefore, that both material and structural cues may be important for children's embodiment, but that future work should systematically investigate their independent or interacting effects.

Our second key finding is that form and movement cues interact for adults but not children. For children, form and movement remain as independent cues to embodiment: even with an asynchronously moving hand, children show some embodiment if it is more human‐like and deemed more task‐effective. This may reflect a shift in strategy—when movement synchrony is absent, children may rely more on task effectiveness than anatomical realism. These findings suggest that children's embodiment is shaped not only by structural familiarity but also by functional relevance and cognitive flexibility. In contrast, adults are limited primarily by movement: when synchrony between observed and felt movement is disrupted, human‐like form alone is not sufficient for eliciting feelings of ownership. These findings imply that the underlying computations for body ownership may differ for children and adults. Recent attempts to model these processes (Chancel et al. 2022) may benefit from considering how adult‐like computations emerge from what may be a different childhood system. Supporting this view, Weijs et al. (2024) found that body ownership across the lifespan is sensitive to temporal mismatches in both visuomotor and tactile domains, with older individuals relying more on top‐down signals. In terms of application, it is important that experiences with adults do not necessarily generalise to those with children: circumstances like asynchronous movement, which typically disrupts embodiment in adults, may still yield embodied experiences for children. This highlights the importance of studying children's experiences per se rather than extrapolating from adults.

We argue that a full understanding of how body ownership is limited or facilitated by visual hand form cannot be achieved without examining longer‐term experiences with new forms. To this end, our second experiment introduced three training sessions where users had the opportunity to build their experience with the Claw form. In line with our hypotheses, we found an increased sense of Ownership for the virtual Claw with training. The only prominent increase, however, was from the first to the second session. While a larger sample size would no doubt increase power to reveal the subtler ongoing increases observed across sessions, it would be unlikely to change the main pattern of initial increased embodiment followed by a plateauing period. This supports the idea that body representations are malleable, with priors changing over time to reflect experience (Apps and Tsakiris 2014), but indicates the heavy weighting users give to prior experience with their existing body and suggests that substantially more experience with a novel body would be needed to overturn these existing priors. In terms of user application in VR games and experiences, our observed pattern of learning suggests that children's sense of bodily self can be rapidly altered by repeated virtual experiences, but over longer periods these changes seem to plateau. This suggests that the sense of bodily self remains robust: the child's fundamental conception of what constitutes their own body is not radically altered by occasional incorporation of a virtual body part. Likewise, we found that training with the Claw had limited impact on embodiment of the other forms. Overall, this could indicate that the risks of long‐term impacts of VR on embodiment are low; however, more research is needed to explore this.

Indeed, responses to our questions on ‘tool‐likeness’ revealed a protective tendency to see virtual forms as tools rather than hands. Tools are in some sense weakly embodied (de Vignemont 2011) or incorporated into the body schema (Cardinali et al. 2009), but they are—at least visually—represented distinctly from body parts (Maimon‐Mor et al. 2020). Across both experiments, responses on experiencing the non‐human virtual forms as a tool were reasonably high. In the first experiment, ratings of Task‐Effectiveness embodiment were higher than ratings of Ownership for children, especially for the Cross form in asynchronous conditions. This suggests that when a form was perceived as useful, children were more willing to treat it as ‘mine’, even without synchrony or resemblance—highlighting a flexible, task‐driven sense of embodiment. Further, in the second experiment, this feeling of Tool‐Likeness increased across sessions specifically for children, but not adults, most notably after the experience of Baseline and Training 1. We therefore conclude that all users were aware of the distinction between a body part and a tool, and that children actively used user experience to sharpen that distinction. It is beyond the scope of the present study to unpick the extent to which this is a core feature of children's body representation, and the extent to which it is a cohort effect of these children living in a technologically mediated world where they must attend to these perceptual lines between the bodily self and technological self (Konca 2022).

The discrepancy between various aspects of the subjective embodiment experience is consistent with the literature (Rohde et al. 2011). We likewise observed in our second experiment increases in Ownership, which stood in contrast to a consistently high degree of Agency. This seemed to depend more on perceived control over the virtual hand form than on its corporeality: across both experiments it was consistently lower for asynchronous than for synchronous conditions. Similarly, high Agency scores appeared to rely on a minimum of actual motor competence. In Experiment 2, although movement fluency improved across sessions for children (see below), Agency was high from the start. Consistent with other work on adults (Mottelson et al. 2023) and children (Dewe et al. 2021), we conclude that visuomotor synchrony is the crucial factor underpinning perceived agency at all ages, while the sense of ownership is also affected by visual hand form.

Alongside the measures of subjective embodiment, we introduced several complementary indices in Experiment 2. First, we examined movement patterns with a virtual hand form as a signature of whether it was incorporated into the body schema. We found that adults were able to move accurately without training. In contrast, for children, the impact of training was striking. Movement times almost halved across the sessions; they shifted from being slower than adults at Baseline to becoming faster than adults Post‐test; and experience with the Claw generalised to the other Hand and Cross forms. This rapid adaption to a new body part implies significant plasticity in children's body schema, consistent with their propensity to use online multisensory information to adapt to a growing body (de Klerk et al. 2021). In applied terms, the findings suggest that young users are well placed to make use of the increasing availability of virtual and mechanical augmentation experiences. However, we note that control over our virtual hand forms was relatively straightforward, involving no new visuomotor mappings such as additional body parts (Kieliba et al. 2021; Won et al. 2015; Krekhov et al. 2019). For more complex control schemes, children may be more limited than adults (Clode et al. 2024), and it is for future work to determine the tolerable level of complexity for young users.

As a final complementary measure of embodiment, we examined margins of error while participants moved across a virtually threatening object (de Vignemont 2023b). We chose a cactus, which we reasoned would induce caution but not fright in our young participants. However, while adults distinguished between this and an equally sized, semantically related neutral object (a flower), children responded similarly. This null result aligns with findings from two studies that assessed skin conductance responses to threats directed at virtual full body (Dewe et al. 2024; Weijs et al. 2021), where some level of response was observed, but it was less intense—potentially reflecting either an attenuated association to embodiment or insufficient salience of the threat. Notably, we did not collect any subjective ratings of perceived threat, which limits our understanding of how participants internalised the threat. In all cases, threats were much milder than those used in adult studies (Braithwaite et al. 2020), or where, for example, movements over a virtual spinning saw (Argelaguet et al. 2016) show sensitivity to both short‐term training and virtual hand form. While defensive reactions are undoubtedly a hallmark of body ownership, we conclude that they prove difficult to index in developmental experiments.

### 4.1 Limitations and Future Directions

Our design had some limitations. In the Catching Feathers task, participants held an Oculus Touch controller, but the virtual forms did not visually depict the controller, and there was no other form of hand tracking (finger movements) or haptic feedback. There may have been some small mismatches between the postural and haptic feedback of the real hand and virtual hand postures). However, we designed the task to minimise the impact of these mismatches in several ways. First, participants were instructed to hold the controller with their palm facing upwards and fingers slightly outstretched, matching the posture of the virtual hand forms. Second, the task involved catching light, floating feathers which were chosen specifically to reduce the expectation of tactile feedback. Third, any postural or haptic mismatch was consistent across all hand form and movement conditions. Therefore, while it may have reduced overall embodiment ratings, it is unlikely to have confounded the relative differences observed between these conditions.

For practical reasons, in Experiment 2, we chose to spread three training sessions over 10 days; this also allowed comparability with children's real‐life VR or gaming experiences. It would be interesting to examine more intense training effects or sessions longer than the 20 minutes we chose as a safe exposure time. Further, while robust enough to reveal some key significant effects, our small sample in Experiment 2 may have limited the detection of more nuanced effects. Due to experimenter error, we were unable to measure the effects of a dual task: this would be of benefit in future work. However, the frequent presentation of the plant challenge (in single‐ and dual‐task conditions) may have contributed to the lack of significant threat responses observed. Finally, while a sense of interaction was gained through the gravitational properties of the feathers’ movements (i.e., they could ‘land’ on the virtual hand form or be manipulated), and while there was minimal perceived discrepancy between predicted (very light) and actual (none) touch, touch cues would likely add to the experience.

In terms of responses, we note that our results are compatible with a metacognitive framework where questionnaire responses reflect a binary judgement of ownership with a confidence judgement layered on top (de Vignemont 2013). Further, recent work suggests that some variance in questionnaire scores results from individual differences in suggestibility (Lush et al. 2020). This has not been shown directly for child samples, and indeed one paper found no relation between children's fantasy‐proneness and their responses to rubber hand illusions (Preston and Kirk 2022). In the present data, responses to our Control question were low, indicating that any general suggestibility effects did not override children's careful and differential responding. We nevertheless advocate for a balanced approach to measuring embodiment: one that accounts for individual differences in suggestibility and developmental variability in how questionnaire‐based measures are interpreted.

## 5 Conclusion

We tested the ability of adults and young children to embody non‐human virtual hand forms. Results reveal that corporeality constrains embodiment at all ages, with less corporeal hands eliciting a lower sense of ownership. For adults, form interacted with movement synchrony, whereas for children of 6–9 years old, these cues operated independently to establish a sense of ownership. We further found that short‐term training increased ownership of a non‐human form at all ages and movement fluency for children. Our research therefore establishes that embodiment of a moving virtual hand operates with somewhat distinct processes for children and adults, and that short‐term training can enhance the feeling that a virtual hand is one's own.

## Author Contributions

**Hayley Dewe**: conceptualisation, data curation, formal analysis, investigation, methodology, project administration, validation, visualisation, writing – original draft, writing – review and editing. **Harry Brenton**: conceptualisation, methodology, resources, software, writing – review and editing. **Isabel Castelow**: data curation, formal analysis, investigation, visualisation. **Emerald Grimshaw**: data curation, formal analysis, investigation, visualisation. **Marco Gillies**: conceptualisation, funding acquisition, methodology, resources, software, validation, writing – review and editing. **Dorothy Cowie**: conceptualisation, data curation, formal analysis, funding acquisition, methodology, project administration, resources, supervision, validation, visualisation, writing – original draft, writing – review and editing.

## Funding

This work was funded by the Economic and Social Research Council (ESRC, ES/P008798/1 awarded to Prof Cowie & Prof Gillies).

## Conflicts of Interest

The authors declare no conflicts of interest.

## Supporting information




**Supporting File 1**: desc70131‐sup‐0001‐SuppMat.docx

## Data Availability

In line with open‐science protocols, the data that support the findings of this study will become openly available here (OSF | VRTrainingFeathers).

## Endnotes

1
https://assetstore.unity.com/packages/3d/characters/humanoids/vrhands‐and‐fp‐arms‐pack‐77815.

2 The two appearance questions (Table 1: Q3A and Q3B) were pooled for analysis because they measured the same underlying concept but were phrased slightly differently to aid participant understanding. Q3A asked whether the participant's own hand looked like the virtual form, and Q3B asked whether the virtual form looked like the participant's own hand.

3 There were no significant differences between the two Apperance questions (Table 1: Q3A and Q3B) on any of the form by movement conditions (all *p*’s > 0.223), and they significantly positively correlated (all *p*’s < 0.001, all *r*’s > 0.49).
