# Supplementary material for: Children's Embodiment of Non‐Human Virtual Hand Forms
Source: Dev Sci. 2026 Jan 20;29(2):e70131. doi: 10.1111/desc.70131 (PMC12819940; doi:10.1111/desc.70131)
Supplement: Supplementary file 1 — Supporting File 1: desc70131‐sup‐0001‐SuppMat.docx [file DESC-29-e70131-s001.docx]

# Supplementary Material

**Table S1**

Information on our final sample sizes for Experiment 1 and Experiment 2 (per measure), explaining drop-out rates.

|  |  | **Children** | **Adults** |
| --- | --- | --- | --- |
| **Experiment 1** | Recruited | 45 | 45 |
|  | Drop-outs | 5 (3 did not like async condition; 2 became distracted) | 0 |
|  | Final sample | 40 | 45 |
|  |  |  |  |
| **Experiment 2** | Recruited | 11 | 12 |
|  | Questionnaires: drop-outs | 1 (no stereo vision) | 1 (experimenter error) |
|  | Questionnaires: final sample | 10 | 11 |
|  | Movement data: drop-outs | 2 (1 no stereo vision; 1 technical error) | 0 |
|  | Movement data: final sample | 9 | 12 |

**Table S2**

Generalisation ratings of Ownership, Agency, Tool-Likeness and Control for the other forms (Hand, Cross, Async Claw) after synchronous Claw-use training in Adults and Children in Experiment 2.

|  | Question | Adults | | Children | |
| --- | --- | --- | --- | --- | --- |
|  |  | Median | IQR | Median | IQR |
| Baseline Claw Async | Ownership | 0.0 | 0.5 | 0.5 | 2.5 |
|  | Agency | 0.0 | 0.5 | 0.0 | 0.8 |
|  | Tool-Likeness | 4.0 | 2.0 | 4.0 | 4.8 |
|  | Control | 0.0 | 1.0 | 1.0 | 2.5 |
| Post-test Claw Async | Ownership | 1.0 | 1.5 | 1.5 | 3.5 |
|  | Agency | 0.0 | 1.0 | 0.0 | 0.8 |
|  | Tool-Likeness | 3.0 | 3.0 | 5.0 | 3.8 |
|  | Control | 1.0 | 1.0 | 1.0 | 1.5 |
| Baseline Cross | Ownership | 2.0 | 4.5 | 4.0 | 2.5 |
|  | Agency | 4.0 | 1.5 | 5.5 | 2.0 |
|  | Tool-Likeness | 5.0 | 1.0 | 4.5 | 2.0 |
|  | Control | 0.0 | 1.0 | 0.0 | 1.0 |
| Post-test Cross | Ownership | 2.0 | 2.5 | 4.5 | 1.8 |
|  | Agency | 4.0 | 1.0 | 4.5 | 2.0 |
|  | Tool-Likeness | 5.0 | 1.5 | 5.0 | 1.8 |
|  | Control | 0.0 | 1.0 | 1.0 | 1.0 |
| Baseline Hand | Ownership | 5.0 | 3.0 | 5.0 | 3.5 |
|  | Agency | 5.0 | 1.0 | 6.0 | 1.0 |
|  | Tool-Likeness | 4.0 | 1.5 | 4.0 | 3.0 |
|  | Control | 0.0 | 0.5 | 0.0 | 1.0 |
| Post-test Hand | Ownership | 5.0 | 2.5 | 5.5 | 3.3 |
|  | Agency | 5.0 | 1.0 | 5.5 | 1.0 |
|  | Tool-Likeness | 4.0 | 1.0 | 5.5 | 1.8 |
|  | Control | 0.0 | 1.0 | 1.0 | 1.0 |

*Note:* Async = Asynchronous movement used for the Claw form only. IQR represents Interquartile range.

**Table S3**

Threat responses for Adults, as the difference between the height above the cactus and the height above the flower obstacles (mm) for each session in Experiment 2.

| Participant | Baseline | Training 1 | Training 2 | Training 3 | Post-test |
| --- | --- | --- | --- | --- | --- |
| 1 | 3.50 | 0.70 | 2.01 | -0.54 | 3.93 |
| 2 | 7.64 | 2.81 | 4.06 | 1.85 | 3.91 |
| 3 | 10.05 | 4.18 | 3.66 | -1.48 | 4.14 |
| 4 | 6.78 | 0.04 | 6.54 | 3.85 | 2.3 |
| 5 | 3.47 | 6.29 | -0.81 | 0.48 | 0.37 |
| 6 | 3.82 | 3.08 | 1.21 | 2.22 | 1.6 |
| 7 | -5.14 | -4.41 | 1.34 | -4.01 | 2.57 |
| 8 | 3.53 | -2.53 | 10.21 | 5.34 | 1.11 |
| 9 | 0.72 | 6.35 | 1.83 | 1.72 | 1.77 |
| 10 | 0.53 | 3.28 | -2.75 | -7.68 | -0.31 |
| 11 | 3.37 | -2.27 | 1.3 | 1.28 | 4.32 |
| 12 | 1.60 | -0.43 | 5.20 | 5.20 | 4.01 |

**Table S4**

Threat responses for Children, as the peak height above the cactus obstacle (mm) for each session in Experiment 2.

| Participant | Baseline | Training 1 | Training 2 | Training 3 | Post-test |
| --- | --- | --- | --- | --- | --- |
| 1 | 16.70 | 22.31 | 12.73 | 25.08 | 24.92 |
| 2 | 26.86 | 24.41 | 21.24 | 24.11 | 26.05 |
| 3 | 31.39 | 23.70 | 25.51 | 26.15 | 21.13 |
| 4 | 30.39 | 34.89 | 32.52 | 34.41 | 31.05 |
| 5 | 13.76 | 23.87 | 13.97 | 17.98 | 13.78 |
| 6 | 25.54 | 26.10 | 20.06 | 18.81 | 13.66 |
| 7 | 23.89 | 13.13 | 12.6 | 12.28 | 14.68 |
| 8 | 19.40 | 25.04 | 18.85 | 21.83 | 27.35 |
| 9 | 17.24 | 9.64 | 18.99 | 16.16 | 23.14 |
|  |  |  |  |  |  |
|  |  |  |  |  |  |
